# Risk factors for COVID-19 associated pulmonary aspergillosis and outcomes in patients with acute respiratory failure in a respiratory sub-intensive care unit

**DOI:** 10.1186/s12879-024-09283-3

**Published:** 2024-04-11

**Authors:** Alessandra Iacovelli, Alessandra Oliva, Flavio Marco Mirabelli, Silvia Giannone, Marianna Laguardia, Matteo Morviducci, Maria Luisa Nicolardi, Emma Repaci, Maria Teresa Sanzari, Cristiana Leanza, Giammarco Raponi, Claudio Mastroianni, Paolo Palange

**Affiliations:** 1 Department of Public Health and Infectious Diseases, Sapienza University of Rome Italy Pulmonology Respiratory and Critical Care Unit, Policlinico Umberto I Hospital Rome, Rome, Italy; 2 Department of Public Health and Infectious Diseases, Sapienza University of Rome, Rome, Italy

**Keywords:** CAPA, COVID-19, Respiratory failure, Sub-intensive care unit, Lymphocytopenia

## Abstract

**Background:**

COVID-19-associated pulmonary aspergillosis (CAPA) is burdened by high mortality. Data are lacking about non-ICU patients. Aims of this study were to: (i) assess the incidence and prevalence of CAPA in a respiratory sub-intensive care unit, (ii) evaluate its risk factors and (iii) impact on in-hospital mortality. Secondary aims were to: (i) assess factors associated to mortality, and (ii) evaluate significant features in hematological patients.

**Materials and methods:**

This was a single-center, retrospective study of COVID-19 patients with acute respiratory failure. A cohort of CAPA patients was compared to a non-CAPA cohort. Among patients with CAPA, a cohort of hematological patients was further compared to another of non-hematological patients.

**Results:**

Three hundred fifty patients were included in the study. Median P/F ratio at the admission to sub-intensive unit was 225 mmHg (IQR 155–314). 55 (15.7%) developed CAPA (incidence of 5.5%). Eighteen had probable CAPA (37.3%), 37 (67.3%) possible CAPA and none proven CAPA. Diagnosis of CAPA occurred at a median of 17 days (IQR 12–31) from SARS-CoV-2 infection. Independent risk factors for CAPA were hematological malignancy [OR 1.74 (95%CI 0.75–4.37), *p* = 0.0003], lymphocytopenia [OR 2.29 (95%CI 1.12–4.86), *p* = 0.02], and COPD [OR 2.74 (95%CI 1.19–5.08), *p* = 0.014]. Mortality rate was higher in CAPA cohort (61.8% vs 22.7%, *p* < 0.0001). CAPA resulted an independent risk factor for in-hospital mortality [OR 2.92 (95%CI 1.47–5.89), *p* = 0.0024]. Among CAPA patients, age > 65 years resulted a predictor of mortality [OR 5.09 (95% CI 1.20–26.92), *p* = 0.035]. No differences were observed in hematological cohort.

**Conclusion:**

CAPA is a life-threatening condition with high mortality rates. It should be promptly suspected, especially in case of hematological malignancy, COPD and lymphocytopenia.

**Supplementary Information:**

The online version contains supplementary material available at 10.1186/s12879-024-09283-3.

## Introduction

Invasive pulmonary aspergillosis (IPA) has been observed in association with respiratory viral illnesses, such as influenza, SARS-CoV-1, and MERS [1–3].

Nowadays, COVID-19-associated pulmonary aspergillosis (CAPA) has been recognized as a major complication of critically ill COVID-19 patients [4]. According to ECMM/ISHAM, CAPA is classified into possible, probable, and proven [5]. It is defined as IPA developing subsequent to SARS-CoV-2 infection and may be suspected in case of i) refractory fever for more than 3 days or new onset fever after a period of defervescence lasting longer than 48 h during appropriate antibiotic therapy, ii) worsening respiratory status despite receiving all recommended treatments for COVID-19; iii) hemoptysis; and iv) pleural friction rub or chest pain [5].

CAPA has been reported mainly in intensive care unit (ICU), affecting up to 10%-20% of COVID-19 patients [6, 7]. Incidence rates may vary from 5 to 40% across different geographic regions, with higher rates observed in patients requiring mechanical ventilation [8]. CAPA is a life-threatening condition, with high mortality rates, usually exceeding 40–60% even with appropriate antifungal treatment [7, 9–11]. Previously reported risk factors included age, chronic respiratory diseases, chronic renal failure, chronic corticosteroid use, neutropenia, lymphopenia, severe COVID-19 requiring mechanical ventilation and tocilizumab administration [7, 8, 12–16].

Diagnosis remains challenging due to the lack of strong consensus definitions and because clinical and radiological findings can mimic those of severe COVID-19 [5, 17–19].

Furthermore, data are lacking concerning non-ICU patients and possible clinical differences between hematological patients and non-hematological patients with CAPA.

Aims of this study were to (i) assess the incidence and prevalence of CAPA patients hospitalized in a respiratory sub-intensive care unit, (ii) evaluate the risk factors for CAPA development and (iii) examine the impact of CAPA on in-hospital mortality. Among the CAPA cohort, secondary aims were to (i) assess factors independently associated with mortality, (ii) evaluate clinical differences between hematological and non-hematological patients.

## Materials and methods

### Study design

We conducted a single-center, retrospective study on patients with COVID-19 pneumonia and respiratory failure hospitalized in a respiratory sub-intensive care unit at Azienda Ospedaliero-Universitaria Policlinico Umberto I, Sapienza University of Rome, from January 2021 to December 2022. A cohort of CAPA patients was compared to a cohort of non-CAPA patients. Among patients with CAPA, a cohort of hematological patients was further compared to another of non-hematological ones.

Inclusion criteria were: (i) diagnosis of COVID-19 pneumonia and respiratory failure and/or acute respiratory distress syndrome (ARDS), (ii) hospitalization in the respiratory sub-intensive care unit for > 48 h and (iii) age > 18 years. Exclusion criteria included: age < 18 years, hospitalization in the respiratory sub-intensive care unit for < 48 h and missing data.

The study received approval from the local Ethics Committee (ID Prot. 109/2020).

### Setting

Starting from September 2020, we set up a respiratory sub-intensive care unit with 42 beds.

In our respiratory sub-intensive care unit patients were admitted in case of acute respiratory failure and/or ARDS due to COVID-19 pneumonia, requiring oxygen therapy and/or Helmet continuous positive airway pressure (CPAP) treatment or non-invasive mechanical ventilation (NIV). In patients with tracheostomy, invasive mechanical ventilation (IMV) was employed.

Patients required the use of continuous vital signs monitoring, and, in most cases, central venous catheter (CVC) or arterial catheters’ placement, total parenteral nutrition and, in case of non-adaptation to ventilation, sedation. We administered dexmedetomidine for sedation, or morphine or midazolam as a secondary line in cases of inadequate response to dexmedetomidine.

Transfer to ICU was required if patients needed orotracheal intubation and IMV.

### Clinical criteria and microbiological methods for diagnostic cultures and infection management

CAPA was defined according to recently proposed definitions [5] as well as practice guidelines [20] using a combination of clinical, radiological, and mycological features of the disease.

Respiratory samples included specimens such us tracheobronchial aspirate (TBA) and/or broncolavage (BAL) (when feasible) and were collected on clinical criteria. Bronchoscopy was not routinely performed and was deemed unfeasible, due to technical difficulties with performing an invasive exam in patients with severe respiratory failure who required CPAP and/or NIV. On respiratory samples, galactomannan (GM) and fungal culture were performed. Fungal cultures were incubated for 7 days at 30 °C on Sabouraud selective media, whereas GM test in serum, BAL and TBA was performed according to manufacturer’s instructions (Platelia Aspergillus EIA, Bio-Rad).

In case of suspected CAPA, the clinical approach was managed together with a dedicated infectious disease specialist (author name, AO). When feasible, chest CT scan was repeated to detect lesions compatible with IPA and was analyzed by dedicated pneumologist and radiologist. In instances of uncertainty, a panel discussion was conducted.

### Definitions

Respiratory failure was diagnosed for PaO_2_ values < 60 mmHg at room air at arterial blood gases (ABGs) upon admission to our ward or to the emergency department, whereas PaO_2_/ FiO_2_ ratio (P/F ratio) was used as an indicator of severity, according to Berlin definitions [21]. Only the P/F ratio at admission was included in the statistical analysis. Diagnosis of COVID-19 pneumonia relied on clinical data, ABGs and chest CT scan performed for all patients at hospital admission [22]. Severe and critical disease were defined according to WHO definitions [23].

Prior (30-day) infections referred to infections diagnosed within 30 days before admission; prior (30-day) antibiotic exposure included receiving antibiotic therapy in the 30 days preceding the diagnosis of CAPA. Chronic steroid treatment was defined as the use of prednisone or its equivalent at a dosage of at least 0.5 mg/kg/day for a minimum of 30-days before admission. Immunodeficiency was defined as the presence of primitive or secondary immunodeficiency conditions (e.g., AIDS, active chemotherapy) [24].

Lymphocytopenia was diagnosed at respiratory sub-intensive care unit admission if the lymphocyte count was < 750 cells × 10^3^/mm^3^ [25].

APACHE II and Charlson Comorbidity Index (CCI) were collected as severity scores at admission.

All patients received a 10-day course of dexamethasone treatment at a dosage of 6 mg/daily due to COVID-19 severity (even patients initially presenting with moderate COVID-19 at admission worsened to a severe stage) and/or antiviral/monoclonal therapy according to available guidelines during the study period [26]. Additionally, all patients received antithrombotic prophylaxis with enoxaparin.

Mortality referred to in-hospital death for all causes.

### Statistical analysis

The data were presented as medians with interquartile ranges (IQR) for continuous variables and as simple frequencies, proportions, and percentages for categorical variables. Mann–Whitney test was used for unpaired samples. Dichotomous variables were compared using Fisher’s exact tests or chi-square test statistics, as appropriate. Survival was analyzed via Kaplan–Meier curves and the statistical significance of differences between the two groups was assessed using the log-rank test. Multivariable logistic regression was conducted to identify independent predictors for CAPA development and for mortality. All statistical analyses were performed using Graph Pad Prism version 10.0.3.

## Results

### General population

The study comprised 350 patients, with a median age of 73 years (IQR 62–83). Among them, 262 (74.8%) presented with severe or critical COVID-19 pneumonia with a median P/F ratio at admission of 225 mmHg (IQR 155–314). General characteristics of the study population are outlined in Table 1.

**Table 1:** Baseline characteristics of patients

	**Total population** ***n* = 350**	**not CAPA cohort** ***n* = 295**	**CAPA cohort** ***n* = 55**	***p*-value**
Age, median (IQR), years	73 (62–83)	72 (61–83)	78 (70–84)	**0.03**
Sex (M), n (%)	225 (64.3)	184 (62.3)	41 (74.5)	0.08
*Demographics*				
Diabetes, n (%)	79 (22.5)	63 (21.3)	16 (29.1)	0.22
Cardiovascular diseases, n (%)	105 (30)	82 (27.8)	23 (41.8)	0.05
Hypertension, n (%)	208 (59.4)	168 (56.9)	40 (72.7)	**0.03**
Chronic respiratory failure, n (%)	28 (8)	19 (6.4)	9 (16.4)	**0.02**
Pulmonary fibrosis, n (%)	15 (4.3)	11 (3.7)	4 (7.3)	0.27
Asthma n (%)	12 (3.4)	11 (3.7)	1 (1.8)	0.7
COPD, n (%)	81 (23.1)	6 (20.3)	21 (38.2)	**0.008**
Bronchiectasis, n (%)	15 (4.3)	10 (3.4)	5 (9.1)	0.07
Chronic hepatopathy, n (%)	5 (1.4)	5 (1.7)	0 (0)	> 0.99
Hematological malignancy, n (%)	34 (9.7)	14 (4.7)	20 (36.4)	** < 0.0001**
Hematological malignancy on active CT treatment, n (%)	22 (6.3)	7 (2.4)	15 (27.3)	** < 0.0001**
Chronic kidney disease, n (%)	50 (14.3)	35 (11.9)	15 (27.3)	**0.0055**
Renal replacement, n (%)	17 (4.8)	13 (4.4)	4 (7.3)	0.32
Obesity (BMI > 30), n (%)	44 (12.5)	35 (11.9)	9 (16.4)	0.37
Autoimmune disease, n (%)	16 (4.6)	14 (4.7)	2 (3.6)	> 0.99
Immunodeficiency, n (%)	37 (10.6)	19 (6.4)	18 (32.7)	** < 0.0001**
Hematopoietic stem cells transplant, n (%)	1 (0.3)	1 (0.34)	0 (0)	> 0.99
Solid organ transplant, n (%)	7 (2)	5 (1.7)	2 (3.6)	0.30
CCI, median (IQR)	5 (3–6)	4 (2–6)	6 (5–8)	** < 0.0001**
Apache II, median (IQR)	9 (6–13)	9 (6–12)	12 (10–17)	** < 0.0001**
Prior (30-d) infections, n (%)	38 (10.8)	29 (9.8)	9 (16.4)	0.15
Prior (30-d) chemotherapy, n (%)	29 (8.2)	13 (4.4)	16 (29.1)	** < 0.0001**
Prior (30-d) antibiotic therapy, n (%)	114 (32.5)	94 (31.9)	20 (36.4)	0.53
Chronic steroid, n (%)	51 (14.6)	33 (11.2)	18 (32.7)	**0.0002**
Anti-CD20 therapy, n (%)	9 (2.6)	0 (0)	9 (16.4)	** < 0.0001**
Lymphocytopenia^a^ at admission, n (%)	184 (52.5)	114 (48.8)	40 (72.7)	**0.0012**
Lymphocytes at admission (× 10^3^/mm^3^), median (IQR)	790 (540–1160)	850 (560–1200)	620 (360–850)	** < 0.001**
Severe or critical COVID-19^b^	262 (74.8)	214 (72.5)	48 (87.3)	**0.02**
PaO_2_/FiO_2_ ratio at admission, median (IQR)	225 (155–314)	223 (155–314)	258 (153–323)	0.80
PaO_2_ at admission, median (IQR)	88 (74–106)	88 (74–106)	85 (68–106)	0.22
FiO_2_ at admission, median (IQR)	40 (28–60)	40 (28–60)	40 (21–60)	0.45
Length of in-hospital stay, median (IQR), days	17 (11–28)	16 (11–25)	30 (19–56)	** < 0.0001**
Transfer to ICU for need to IMV, n (%)	13 (3.7)	7 (2.4)	6 (10.9)	** < 0.0001**
Mortality, n (%)	101 (28.8)	67 (22.7)	34 (61.8)	** < 0.0001**
*Respiratory failure treatment at admission*				
HFNC, n (%)	17 (4.8)	11 (3.7)	6 (10.9)	0.03
Venturi mask, n (%)	225 (64.2)	189 (64.1)	36 (65.45)	0.84
Helmet CPAP, n (%)	41 (11.7)	38 (12.8)	3 (5.4)	0.17
NIV, n (%)	8 (2.2)	4 (1.3)	4 (7.3)	**0.02**
*COVID-19 therapy*				
Remdesivir, n (%)	213 (60.8)	178 (60.3)	35 (63.6)	0.76
Anti-IL6, n (%)	11 (3.1)	6 (2.0)	5 (9.1)	**0.02**
Monoclonal antibody, n (%)	30 (8.5)	15 (5.1)	15 (27.3)	** < 0.0001**

*COPD* chronic obstructive pulmonary disease, *BMI* body mass index, *CCI* Charlson Comorbidity Index, *paO*_*2*_ arterial oxygen tension, *FiO*_*2*_ fraction of inspired oxygen, *ICU* intensive care unit, *IMV* invasive mechanical ventilation, *HFNC* high flow nasal cannula, *CPAP* continuous positive airways pressure, *NIV* non-invasive mechanical ventilation

^a^Lymphocytopenia is defined as lymphocytes count inferior to 750 cells × 10^3^/mm^3^

^b^Sever or critical COVID-19 was defined according to WHO definitions [23]

Most patients (184, 52.5%) presented lymphocytopenia, with median lymphocyte count of 790 cells × 10^3^/mm^3^ (IQR 540–1160). The overall mortality rate was 28.8%.

The general population was further divided into CAPA and non-CAPA cohorts (Table 1). Table 2 shows specific features of CAPA cohort.

**Table 2:** Characteristics of CAPA cohort

CAPA cohort (*n* = 55)	
Days from SARS-CoV-2 infection to diagnosis of CAPA, median (IQR)	17 (12–31)
Days from clinical worsening to diagnosis of CAPA, median (IQR)	3 (0–6)
Days to diagnosis of CAPA to death, median (IQR)	6 (4–15)
Post-mortem diagnosis, n (%)	5 (9.1)
PaO_2_/FiO_2_ ratio at diagnosis of CAPA, median (IQR)	127 (88.5–200.8)
Classification of CAPA	
*Proven, n (%)*	0 (0)
*Probable, n (%)*	18 (32.7)
*Possible, n (%)*	37 (67.3)
Respiratory treatment at diagnosis of CAPA	
*Venturi Mask, n (%)*	15 (27.3)
*HFNC, n (%)*	23 (41.8)
*Helmet CPAP, n (%)*	6 (10.9)
*NIV, n (%)*	6 (10.9)
Clinical criteria	
*Fever, n (%)*	13 (23.6)
*Worsening respiratory failure, n (%)*	52 (94.5)
*Haemoptisis, n (%)*	6 (10.9)
Radiological criteria^a^ *n* = 28	
*Lung infiltrates, n (%)*	25 (89.3)
*Cavitations, n (%)*	1 (3.6)
*Nodules, n (%)*	6 (21.4)
Microbiological criteria	
*Aspergillus spp growth, n (%)*	15 (27.3)
*Respiratory samples Galactomannan index, median (IQR)*^*b*^	3.75 (1.9–6.5)
*Serum Galactomannan index, median (IQR)*^*c*^	1.45 (0.75–3.97)
Antifungal therapy^d^ *n* = 50	
*Voriconazole, n (%)*	4 (8.0)
*Isavuconazole, n (%)*	40 (80.0)
*Amphotericin B, n (%)*	6 (12.0)

*PaO*_*2*_ arterial oxygen tension, *FiO*_*2*_ fraction of inspired oxygen, *HFNC* high flow nasal cannula, *CPAP* continuous positive airways pressure, *NIV* non-invasive mechanical ventilation

^a^Chest CT was performed only in 28 patients

^b^2 BAL, 53 TBA

^c^6 samples

^d^In 5 cases CAPA was diagnosed post-mortem so patients did not received any treatment

### CAPA cohort

As shown in Table 2, 55 (15.7%) patients developed CAPA, with an incidence of 5.5% over the two years of observation.

There were no cases of proven CAPA, 18 (32.7%) were classified as probable CAPA and 37 (67.3%) as possible CAPA [5]. Mycological features of CAPA cohort are reported in Supplementary Table 1.

The diagnosis of probable/possible CAPA was established at a median of 17 days (IQR 12–31) from SARS-CoV-2 infection and at a median of 3 days (IQR 0–6) from the onset of CAPA symptoms. In 5 (9.1%) cases, the diagnosis occurred post-mortem. Mortality rate was 61.8% (Table 1). Death occurred at a median of 6 days (IQR 4–15) from CAPA diagnosis. At the time of CAPA diagnosis, most patients presented with severe respiratory failure with a median P/F ratio of 127 (IQR 88.5–200.8).

Most patients (52, 94.5%) presented with worsening respiratory failure. *Aspergillus* spp growth was observed only in 15 (27.3%) respiratory specimens, with *Aspergillus fumigatus* being the most common species. A new chest CT scan was repeated in 28 (50.9%) patients. Main radiological findings suggestive for CAPA included new lung infiltrates (25, 89.3%) and nodules (6, 21.4%).

Isavuconazole was administered in 80% of patients as the main choice for antifungal treatment.

### Comparison between CAPA and non-CAPA cohort

As depicted in Table 1, patients with CAPA presented a high rate of severe or critical COVID-19 at admission (87.3% vs 72.5%, *p* = 0.02) and were more likely to receive NIV treatment compared to non-CAPA patients (7.3% vs 1.3%, *p* = 0.02). They were also older [78 (70–84) vs 72 (61–83) years, *p* = 0.03] and had higher Charlson Comorbidity Index (CCI) and APACHE II index (*p* < 0.0001). Patients with CAPA had a higher rate of lymphocytopenia at admission (72.7% vs 48.8%, *p* = 0.0012**)** with a lower median lymphocyte count [620 (360–850) vs 850 (560–1200) cells × 10^3^/mm^3^, *p* < 0.001].

COPD was more common in the CAPA cohort (38.2% vs 20.3%, *p* = 0.008).

Chronic corticosteroids therapy (32.7% vs 11.2%, *p* = 0.0002), previous anti-CD20 treatment (16.4% vs 0%, *p* < 0.0001) and chemotherapy in the 30-d prior to CAPA development (29.1% vs 4.4%, *p* < 0.0001) were more common in the CAPA cohort. Although not statistically significant, prior 30-day infections rate was higher in CAPA cohort (16.4% vs 9.8%, *p* = 0.15).

According to their worse COVID-19 condition, CAPA patients received more commonly anti-IL6 and monoclonal antibody [(9.1% vs 2.0% and 27.3% vs 5.1%, *p* = 0.02 and *p* < 0001), respectively].

The discrepancy in the number of patients transferred to the ICU differed between the two cohorts and was statistically significant [7 (2.4%) vs 6 (10.9%) (*p* < 0.0001)]. Length of stay was higher in CAPA cohort [30 (19–56) vs 16 (11–25) days, *p* < 0.0001], as well as mortality rate (61.8% vs 22.7%, *p* < 0.0001).

At multivariable analysis, independent risk factors for CAPA were hematological malignancy [OR 1.74 (95%CI 0.75–4.37), *p* = 0.0003], lymphocytopenia [OR 2.29 (95% CI 1.12–4.86), *p* = 0.02], and COPD [OR 2,74 (95% CI 1.19–5.08), *p* = 0.014] (Table 3).

**Table 3:** Multivariate analyses evaluating risk factors for CAPA development (panel A) and mortality in the overall population (panel B) and in the CAPA cohort (panel C)

*Panel A. Risk factors for CAPA*	OR (CI95%)	*p*-value
Age > 65 years	1.74 (0.75–4.37)	0.21
Hematological malignancy	5.93 (2.26–15.78)	**0.0003**
Lymphocytopenia^a^	2.29 (1.12–4.86)	**0.02**
Severe or critical COVID-19^b^	1.91 (0.81–5.19)	0.16
COPD	2.47 (1.19–5.08)	**0.014**
Immunodeficiency	2.19 (0.79–5.84)	0.123
Chronic steroid therapy	2.38 (0.98–5.60)	0.05
Anti-IL6 therapy	2.70 (0.48–13.17)	0.23
*Panel B. Risk factors for mortality in overall population*	**OR (CI95%)**	***p*-value**
Severe or critical COVID-19^b^	3.59 (1.67–8.27)	**0.0014**
CAPA	2.92 (1.47–5.89)	**0.0024**
Male sex	0.94 (0.52–1.69)	0.8284
Lymphocytopenia^a^	3.23 (1.79–5.97)	**0.0001**
APACHE II > 9	5.55 (2.97–10.70)	** < 0.0001**
CCI > 5	1.59 (0.87–2.91)	0.1292
*Panel C. Risk factors for mortality in CAPA cohort*	**OR (CI95%)**	***p*-value**
APACHE II > 9	2.28 (0.60–8.89)	0.224
Age > 65 years	5.09 (1.20–26.92)	**0.035**

*COPD* chronic obstructive pulmonary disease, *APACHE II* acute physiology and chronic health evaluation II,

*CCI* Charlson Comorbidity Index

^a^Lymphocytopenia is defined as lymphocytes count inferior to 750 cells × 10^3^/mm^3^

^b^Sever or critical COVID-19 was defined according to WHO definitions [23]

### Comparison between survivors and non-survivors in general population

As shown in Table 4, patients who died were older and presented a higher prevalence of comorbidities compared to survivors (*p* < 0.0001). Moreover, non-survivors had a higher APACHE II on admission to the sub-intensive care unit (*p* < 0.0001) and a greater percentage of severe or critical COVID-19 (90.1% vs 68.7%, *p* > 0.0001), requiring a higher median administered FiO_2_ [60% (40–65) vs 35% (24–60), *p* < 0.0001] and mechanical ventilation, including Helmet CPAP and NIV [(18.8% vs 8.8%, *p* = 0.016) and (4.9% vs 1.2%, *p* = 0.047), respectively].

**Table 4:** Comparison between survivors and non-survivors in general population and in CAPA cohort

*General Population*	Survivors *n* = 249 (71.2%)	Non survivors *n* = 101 (28.8%)	*p*-value
CAPA, n (%)	21 (8.4)	34 (33.7)	** < 0.0001**
Age, median (IQR), years	70 (59–78)	83 (73–87)	** < 0.0001**
Sex (M), n (%)	158 (63.4)	67 (66.3)	0.62
*Demographics*			
Diabetes, n (%)	55 (22.1)	24 (23.8)	0.77
Cardiovascular diseases, n (%)	61 (24.5)	44 (43.5)	**0.0008**
Hypertension, n (%)	138 (55.4)	70 (69.3)	**0.01**
Chronic respiratory failure, n (%)	18 (7.2)	10 (9.9)	0.39
Pulmonary fibrosis, n (%)	9 (3.6)	6 (5.9)	0.38
Asthma n (%)	11 (4.4)	1 (0.9)	0.19
COPD, n (%)	50 (20.1)	31 (30.7)	**0.03**
Bronchiectasis, n (%)	9 (3.6)	6 (5.9)	0.38
Chronic hepatopathy, n (%)	4 (1.6)	1 (0.9)	> 0.99
Hematological malignancy, n (%)	17 (6.8)	17 (16.8)	**0.0085**
Chronic kidney disease, n (%)	29 (11.6)	21 (20.8)	**0.04**
Renal replacement, n (%)	11 (4.4)	6 (5.9)	0.58
Obesity (BMI > 30), n (%)	33 (9.4)	11 (10.9)	0.60
Autoimmune disease, n (%)	14 (5.6)	2 (1.9)	0.17
Immunodeficiency, n (%)	24 (9.6)	13 (12.8)	0.44
Hematopoietic stem cells transplant, n (%)	1 (0.4)	0 (0)	> 0.99
Solid organ transplant, n (%)	6 (2.41)	1 (0.9)	0.68
CCI, median (IQR)	4 (2–6)	6 (5–8)	** < 0.0001**
Apache II, median (IQR)	8 (5–11)	12 (10–17)	** < 0.0001**
Prior (30-d) infections, n (%)	16 (6.4)	22 (21.8)	** < 0.0001**
Prior (30-d) chemotherapy, n (%)	14 (5.6)	15 (14.8)	**0.0088**
Prior (30-d) antibiotic therapy, n (%)	69 (27.7)	45 (44.5)	**0.0036**
Chronic steroid, n (%)	34 (13.6)	17 (16.8)	0.50
Anti-CD20 therapy, n (%)	5 (2.0)	4 (3.9)	0.28
Lymphocytopenia^a^ at admission, n (%)	107 (43.0)	77 (76.2)	** < 0.0001**
Lymphocytes at admission (× 10^3^/mm^3^), median (IQR)	930 (610–1265)	570 (390–795)	** < 0.0001**
Severe or critical COVID-19**	171 (68.7)	91 (90.1)	** < 0.0001**
PaO_2_/FiO_2_ ratio at admission, median (IQR)	250 (183–334)	153 (108–265.5)	** < 0.0001**
PaO_2_ at admission, median (IQR)	91 (76.5–108)	80 (67.5–102)	**0.0011**
FiO_2_ at admission, median (IQR)	35 (24–60)	60 (40–65)	** < 0.0001**
Length of in-hospital stay, median (IQR), days	18 (12–27)	17 (9.5–30)	0.31
Transfer to ICU for need to IMV, n (%)	4 (1.6)	9 (8.9)	**0.0025**
*Respiratory failure treatment at admission*			
HFNC, n (%)	11 (4.4)	6 (5.9)	0.58
Venturi mask, n (%)	160 (64.2)	65 (64.4)	> 0.99
Helmet CPAP, n (%)	22 (8.8)	19 (18.8)	**0.016**
NIV, n (%)	3 (1.2)	5 (4.9)	**0.047**
*COVID-19 therapy*			
Remdesivir, n (%)	161 (64.6)	52 (51.5)	**0.03**
Anti-IL6, n (%)	7 (2.8)	4 (3.9)	0.73
Monoclonal antibody, n (%)	19 (63.3)	11 (10.9)	0.39
*CAPA Cohort*	**Survivors** ***n* = 21 (38.2%)**	**Non survivors** ***n* = 34 (61.8%)**	***p*-value**
Age, median (IQR), years	72 (62–80)	78 (72–85)	**0.03**
Sex (M), n (%)	15 (71.4)	26 (76.5)	0.75
*Demographics*			
Diabetes, n (%)	9 (42.9)	7 (20.6)	0.12
Cardiovascular diseases, n (%)	7 (35)	16 (47)	0.41
Hypertension, n (%)	16 (76.2)	24 (70.6)	0.76
Chronic respiratory failure, n (%)	3 (14.3)	6 (17.6)	> 0.99
Pulmonary fibrosis, n (%)	2 (9.5)	2 (5.9)	0.63
Asthma n (%)	1 (4.8)	0 (0)	0.38
COPD, n (%)	7 (33.3)	14 (41.2)	0.77
Bronchiectasis, n (%)	2 (9.5)	3 (8.8)	> 0.99
Chronic hepatopathy, n (%)	0 (0)	0 (0)	> 0.99
Hematological malignancy, n (%)	7 (33.3)	13 (38.2)	0.78
Chronic kidney disease, n (%)	7 (33.3)	8 (23.5)	0.53
Renal replacement, n (%)	1 (4.8)	3 (8.8)	> 0.99
Obesity (BMI > 30), n (%)	4 (19)	5 (14.7)	0.72
Autoimmune disease, n (%)	2 (9.5)	0 (0)	0.14
Immunodeficiency, n (%)	8 (38.1)	10 (29.4)	0.56
Hematopoietic stem cells transplant, n (%)	0 (0)	0 (0)	> 0.99
Solid organ transplant, n (%)	1 (4.8)	1 (2.9)	> 0.99
CCI, median (IQR)	6 (5–8)	6 (5–8)	0.71
Apache II, median (IQR)	11.6 (6–14)	15 (10–19)	**0.04**
Prior (30-d) infections, n (%)	3 (14.3)	6 (17.6)	> 0.99
Prior (30-d) chemotherapy, n (%)	6 (28.6)	10 (29.4)	> 0.99
Prior (30-d) antibiotic therapy, n (%)	6 (28.6)	14 (41.2)	0.39
Chronic steroid, n (%)	8 (38.1)	10 (29.4)	0.56
Anti-CD20 therapy, n (%)	5 (23.8)	4 (11.8)	0.28
Lymphocytopenia^a^ at admission, n (%)	15 (71.4)	25 (73.5)	> 0.99
Lymphocytic count, median (IQR)	668 (425–999)	661 (352–820)	0.79
Severe or critical COVID-19^b^	16 (76.2)	32 (94.1)	0.09
PaO_2_/FiO_2_ ratio at admission, median (IQR)	253 (164–345)	233 (138–304)	0.45
paO_2_ at admission, median (IQR)	88 (68–106)	91 (67–106)	0.82
FiO_2_ at admission, median (IQR)	41 (21–60)	47 (28–60)	0.36
Length of in-hospital stay, median (IQR), days	52 (28–68)	29 (13–42)	**0.0006**
Transfer to ICU for need of IMV, n (%)	1 (4.8)	5 (14.7)	0.39
*Respiratory failure treatment at admission*			
HFNC, n (%)	3 (14.3)	3 (8.8)	0.66
Venturi mask, n (%)	14 (66.7)	22 (64.7)	> 0.99
Helmet CPAP, n (%)	0 (0)	3 (8.8)	0.28
NIV, n (%)	0 (0)	4 (11.8)	0.28
*COVID-19 therapy*			
Remdesivir, n (%)	16 (76.2)	19 (55.9)	0.15
Anti-IL6, n (%)	4 (19)	1 (2.9)	0.06
Monoclonal antibody, n (%)	8 (38.1)	7 (20.6)	0.21

*COPD* chronic obstructive pulmonary disease, *BMI* body mass index, *CCI* Charlson Comorbidity Index, *paO*_*2*_ arterial oxygen tension, *FiO*_*2*_ fraction of inspired oxygen, *ICU* intensive care unit, *IMV* invasive mechanical ventilation, *HFNC* high flow nasal cannula, *CPAP* continuous positive airways pressure, *NIV* non-invasive mechanical ventilation

^a^Lymphocytopenia is defined as lymphocytes count inferior to 750 cells × 10^3^/mm^3^

^b^Sever or critical COVID-19 was defined according to WHO definitions [23]

Non-survivors also presented a higher rate of lymphocytopenia (*p* < 0.0001) with significantly lower lymphocyte count [570 (390–795) vs 930 (610–1265) cells × 10^3^/mm^3^, *p* < 0.0001)] than survivors. The development of CAPA was more common in non-survivors (42% vs 14%, *p* = 0.0001).

**Fig. 1:**
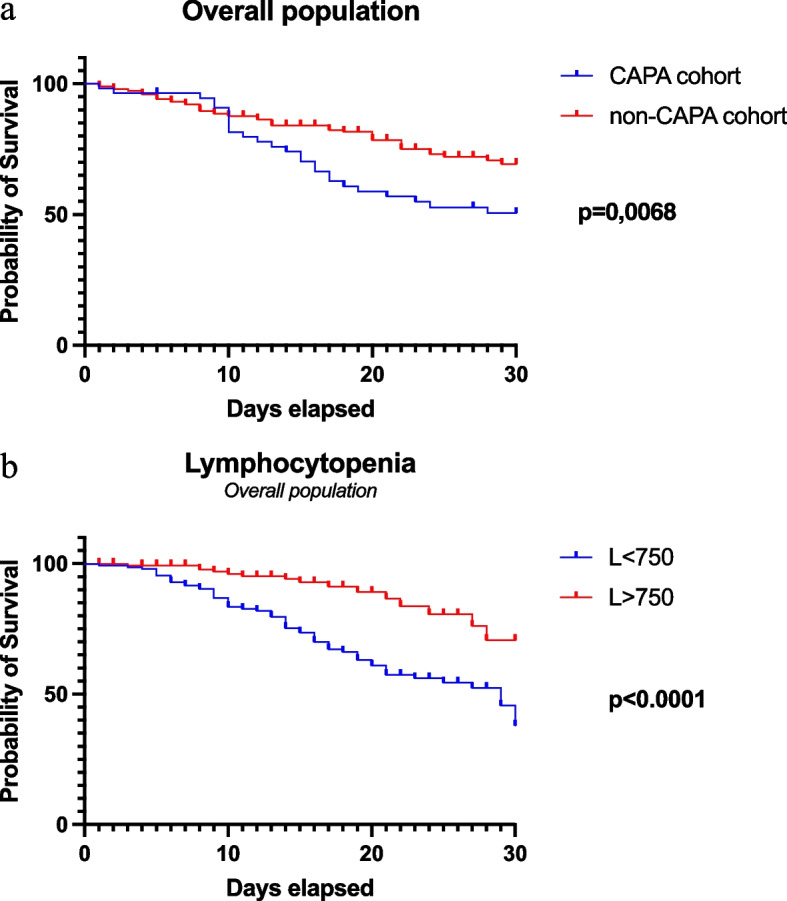
**a**-**b**. Kaplan–Meier survival curves in overall population. **a** Kaplan–Meier survival curves showing differences in survival at 30-d in CAPA cohort versus non-CAPA cohort; (**b**) Kaplan–Meier survival curves showing differences in survival at 30-d according to lymphocytopenia

Kaplan–Meier survival curves have demonstrated different mortality rates in patients with CAPA development and in those with lymphocytopenia (Fig. 1a-b).

At multivariable analysis, CAPA emerged as an independent risk factor for in-hospital mortality [OR 2.92 (95% CI 1.47–5.89), *p* = 0.0024]. Other predictors of mortality were severe or critical COVID-19 at admission to respiratory sub-intensive care unit [OR 3.59 (95% CI 1.67–8.27), *p* = 0.0014], APACHE II > 9 [OR 5.55 (95% CI 2.97–10.70), *p* < 0.0001], and lymphocytopenia [OR 3.23 (95% CI 1.79–5.97), *p* = 0.0001] (Table 3).

### Comparison between survivors and non-survivors in CAPA cohort

In the CAPA cohort, deceased patients were older [78 (72–85) vs 72 (62–80) years, *p* = 0.03] (Table 4). Non-survivors also exhibited a higher APACHE II at admission in sub-intensive care unit (*p* = 0.04). Length of stay was higher in patients who survived (*p* = 0.0006).

At multivariable analysis, only age > 65 years resulted as a predictor of mortality [OR 5.09 (95% CI 1.20–26.92), *p* = 0.035] (Table 3).

### Comparison between hematological and non-hematological patients with CAPA

Non-hematological patients displayed higher prevalence of cardiovascular disease, diabetes, and chronic kidney disease (*p* = 0.02, 0.03 and 0.005, respectively) (Table 5). Conversely, in line with their malignancy condition, they presented a higher percentage of immunodeficiency, prior 30-day chemotherapy and anti-CD20 therapy. Mortality rate was similar between the two cohorts, while the rate of transfer to ICU was higher in the hematological cohort [15% vs 8.6%, *p* = 0.66], albeit not statistically significant.

**Table 5:** Comparison between hematological and non-hematological patients with CAPA

	**Hematologic patients with CAPA** ***n* = 20**	**Non hematologic patients with CAPA** ***n* = 35**	***p*-value**
Age, median (IQR), years	73.5 (66.5–81.75)	78 (71–85)	0.23
Sex (M), n (%)	15 (75)	26 (74.3)	> 0.99
*Demographics*			
Diabetes, n (%)	2 (10)	14 (40)	**0.03**
Cardiovascular diseases, n (%)	4 (20)	19 (54.3)	**0.02**
Hypertension, n (%)	12 (60)	28 (80)	0.13
Chronic respiratory failure, n (%)	2 (10)	7 (20)	0.46
Pulmonary fibrosis, n (%)	2 (10)	2 (5.7)	0.61
Asthma n (%)	0 (0)	1 (2.8)	> 0.99
COPD, n (%)	6 (30)	15 (42.8)	0.40
Bronchiectasis, n (%)	2 (10)	3 (8.6)	> 0.99
Chronic hepatopathy, n (%)	0 (0)	0(0)	> 0.99
Chronic kidney disease, n (%)	1 (5)	14 (40)	**0.005**
Renal replacement, n (%)	1 (5)	3 (8.6)	> 0.99
Obesity (BMI > 30), n (%)	2 (10)	7 (20)	0.46
Autoimmune disease, n (%)	0 (0)	2 (5.7)	0.53
Immunodeficiency, n (%)	13 (65)	5 (14.3)	**0.0002**
Hematopoietic stem cells transplant, n (%)	0 (0)	0 (0)	> 0.99
Solid organ transplant, n (%)	0 (0)	2 (5.7)	0.53
CCI, median (IQR)	5 (4.25–7.75)	6 (5–9)	0.22
Apache II, median (IQR)	12 (9–14.75)	13 (10–19)	0.35
Prior (30-d) infections, n (%)	1 (5)	8 (22.8)	0.13
Prior (30-d) chemotherapy, n (%)	15 (75)	1 (2.9)	** < 0.0001**
Prior (30-d) antibiotic therapy, n (%)	8 (40)	12 (34.3)	0.77
Chronic steroid treatment, n (%)	8 (40)	10 (28.6)	0.55
Anti-CD20 therapy, n (%)	8 (40)	1 (2.9)	**0.0007**
Lymphocytopenia^a^ at admission, n (%)	15 (75)	25 (71.4)	> 0.99
Lymphocytic count, median (IQR)	500 (285–825)	660 (490–850)	0.17
Severe or critical COVID-19^b^	17 (85)	31 (88.6)	0.70
PaO_2_/FiO_2_ ratio at admission, median (IQR)	288.5 (217–336.5)	201 (143–300)	0.10
paO_2_ at admission, median (IQR)	97.5 (76.25–106)	74 (66–99)	0.66
FiO_2_ at admission, median (IQR)	35 (22.75–47.5)	50 (21–60)	0.31
Length of in-hospital stay, median (IQR), days	29.5 (22.25–47.75)	33 (18–58)	0.91
Transfer to ICU for need for IMV, n (%)	3 (15)	3 (8.6)	0.66
Mortality, n (%)	13 (65)	21 (60)	0.78
*Respiratory failure treatment at admission*			
HFNC, n (%)	2 (10)	4 (11.4)	> 0.99
Venturi mask, n (%)	14 (70)	22 (62.9)	0.77
Helmet CPAP, n (%)	0 (0)	3 (8.6)	0.29
NIV, n (%)	1 (5)	3 (8.6)	> 0.99
*COVID-19 therapy*			
Remdesivir, n (%)	17 (85)	18 (51.4)	**0.02**
Anti-IL6, n (%)	10 (50)	5 (14.3)	**0.01**
Monoclonal antibody, n (%)	3 (15)	2 (5.7)	0.34

*COPD* chronic obstructive pulmonary disease, *BMI* body mass index, *CCI* Charlson Comorbity Index, *paO*_*2*_ arterial oxygen tension, *FiO*_*2*_ fraction of inspired oxygen, *ICU* intensive care unit, *IMV* invasive mechanical ventilation, *HFNC* high flow nasal cannula, *CPAP* continuous positive airways pressure, *NIV* non-invasive mechanical ventilation

^a^Lymphocytopenia is defined as lymphocytes count inferior to 750 cells × 10^3^/mm^3^

^b^Sever or critical COVID-19 was defined according to WHO definitions [76]

**Fig. 2:**
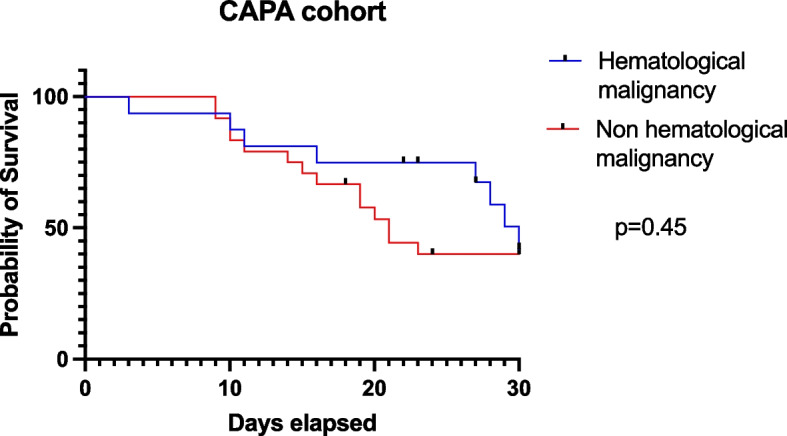
Kaplan–Meier curves in CAPA cohort. Kaplan–Meier curves showing survival at 30-d from the admission according to the presence of hematological malignancy in CAPA cohort

Kaplan–Meier curves at 30 days from admission did not differ in patients with hematological malignancy (Fig. 2).

## Discussion

Our main findings were: i) in patients hospitalized in sub-intensive care unit for severe and/or critical COVID-19, CAPA represents a main complication and a risk factor for mortality even in immunocompetent patients; ii) CAPA is burdened by a high mortality rate, especially within the first days following diagnosis; iii) patients affected by hematological malignancy, COPD and lymphocytopenia are at heightened risk of developing CAPA.

To the best of our knowledge, this is the first study evaluating CAPA in non-ICU patients, reporting original data about a specific respiratory sub-intensive setting of care. Our findings confirm previous data from ICU studies [4, 8]. First, CAPA affected older patients with multiple comorbidities, who presented severe or critical COVID-19 and a higher APACHE II score at admission. Second, we reported a prevalence of CAPA of 15.7%. In 67.3% of cases, we diagnosed possible CAPA, occurring at a median of 17 days from SARS-CoV-2 infection. Third, CAPA was associated with a notably high mortality rate exceeding that of non-CAPA patients (61.8% vs 22.7%, *p* < 0.0001).

Additionally, CAPA emerged as an independent risk factor for in-hospital mortality at multivariate analysis. Kaplan–Meier curves sustained this finding, showing a significant increase of mortality in CAPA patients at 30 days from admission. Remarkably, in most cases, patients died within a median of 6 days from diagnosis, underscoring the severity of the disease despite prompt antifungal therapy. Notably, 5 (9.1%) patients died during samples analysis time, meaning before a correct diagnosis and treatment could have been obtained. This data supports the urgency of early suspicion and diagnosis of CAPA in high-risk patients.

However, prompt diagnosis can be hindered by nonspecific clinical and radiological features of the disease [5]. Indeed, in our cohort, main clinical manifestations included worsening of respiratory failure and fever. Likewise, we performed a chest CT scan only in 50.9% of patients, due to technical difficulties in transporting critically ill patients with rapid respiratory deterioration. Nevertheless, among those scanned, we observed, as main patterns of CAPA, lung infiltrates and nodules, confirming the nonspecific patterns already described in literature.

In this scenario, recognizing risk factors for CAPA is challenging. Previous studies proposed several risk factors [7, 12–16, 27], such as older age, tocilizumab treatment and the need for mechanical ventilation. A recent study [4], confirmed tocilizumab as a risk factor for probable CAPA, together with COPD and prior 28-d steroid therapy. In our study tocilizumab treatment was more common in patients who developed CAPA (*p* = 0.02), especially if affected by hematological malignancy (*p* = 0.01), but it did not represent an independent predictor of CAPA. Likewise, the need for mechanical ventilation did not result as an independent risk factor for CAPA, although administered more commonly to patients who died, in line with the severity of their COVID-19 pneumonia related condition.

In our multivariate logistic regression model, independent risk factors for CAPA development were hematological malignancy, COPD and lymphocytopenia (e.g., lymphocytes < 750 cells × 10^3^/mm^3^ at admission).

Hematological malignancies are traditionally considered a risk factor for IPA [28] and were more common in non-survivors in our study. Nevertheless, we did not observe significant differences in hematological cohort beyond factors related to hematological condition itself. In other terms, this could mean that mortality in CAPA cohort could be related only to CAPA and severe COVID-19. Indeed, Kaplan–Meier curves did not show a different probability of survival between hematological and non-hematological patients and at multivariate analysis, only age > 65 years was found to be an independent predictor of mortality in CAPA cohort.

COPD has been recently considered as an emerging risk factor for IPA, in patients on chronic corticosteroid therapy [29]. Chronic steroid treatment was more common in CAPA cohort but it did not predict CAPA development at multivariate analysis. Regarding the use of dexamethasone, recent evidence suggested that it increases the risk of CAPA [30, 31], so its administration could have represented a possible risk factor in our cohort. Nevertheless, we did not investigate the role of dexamethasone since all patients received this treatment. Interestingly, our cohort included COPD patients at any stage of the disease and with any treatment regimen suggesting that COPD itself can be a risk factor for CAPA regardless of chronic steroid treatment, in line with previous studies [15, 16].

An important finding of our study is the association of lymphocytopenia with poor prognosis. Several authors already reported lymphocytopenia as a risk factor for CAPA [32, 33] and as a predictor of severity in COVID-19 patients [9]. In our study, lymphocytopenia is a predictor of poor prognosis and correlate both with mortality in overall COVID-19 population and CAPA development. Kaplan–Meier curves at 30-days showed a significant increase in mortality in patients with lymphocytopenia. As a matter of interest, in the CAPA population, no differences in terms of lymphocytopenia are observed between survivors and non-survivors. This suggests that in most patients lymphocytopenia could be caused by SARS-CoV-2 infection [30, 34]. Consequently, CAPA could develop also in immunocompetent patients who experience a transient immunocompromise condition [32]. Indeed, severe COVID-19 is known to decrease the number and functionality of CD4 + T and CD8 + T-cells and induce a hyperinflammatory state that enhances fungal growth [32–34]. Lymphocytopenia has also been already identified as a predictor of influenza associated pulmonary aspergillosis (IAPA) [35]. Moreover, data from lung transplant recipients reported a high mortality in patients affected by respiratory viral illnesses and IPA superinfection [31]. These evidences suggest that a possible relationship between other respiratory viruses and secondary fungal infections should be considered and further investigated.

Our study has some limitations. It is a single-center, retrospective study. Patients in both cohorts were admitted in different pandemic periods. Variations in the pathogenicity of different SARS-CoV-2 variants and the improvement in medical staff experience and treatment efficacy over time, may have influenced patient outcomes. Presented data reflect a real-life scenario with no predefined CAPA screening protocol and diagnosis was mainly based on clinical suspicion. Moreover, bronchoscopy was not routinely performed and, accordingly, we mainly diagnosed possible rather than probable CAPA. We acknowledge that using the ECMM/ISHAM diagnostic criteria may be limited by the fact that our patients were not hospitalized in the ICU at the moment of CAPA. However, since specific guidelines for CAPA in sub-intensive care units are lacking, we were forced to rely on guidelines that apply to the setting most similar to ours, namely the ICU setting. Finally, not all patients repeated chest CT scan at clinical worsening.

In conclusion, CAPA is a life-threatening condition in patients hospitalized in respiratory sub-intensive care unit for severe COVID-19, even among immunocompetent patients. Given its high short-term mortality rate, CAPA should be promptly suspected in patients experiencing respiratory worsening despite appropriate COVID-19 treatment, especially in those affected by hematological malignancies, COPD and lymphocytopenia.

### Supplementary Information

**Supplementary Material 1**.

## Data Availability

Data are available upon request from corresponding author.

## Abbreviations

IPA: Invasive pulmonary aspergillosis
CAPA: COVID-19-associated pulmonary aspergillosis
ICU: Intensive care unit
ARDS: Acute respiratory distress syndrome
NIV: Non-invasive mechanical ventilation
IMV: Invasive mechanical ventilation
CVC: Central venous catheter
TBA: Tracheobronchial aspirate
BAL: Broncolavage
GM: Galactomannan
P/F ratio: PaO_2_/ FiO_2_ ratio
COPD: Chronic obstructive pulmonary disease
IAPA: Influenza associated pulmonary aspergillosis

## References

[1] Lat A, Bhadelia N, Miko B (2010). Invasive aspergillosis after pandemic (H1N1) 2009. Emerg Infect Dis.

[2] Shah MM, Hsiao EI, Kirsch CM (2018). Invasive pulmonary aspergillosis and influenza co-infection in immunocompetent hosts: case reports and review of the literature. Diagn Microbiol Infect Dis..

[3] Milne-Price S, Miazgowicz KL, Munster VJ (2014). The emergence of the Middle East respiratory syndrome coronavirus. Pathog Dis..

[4] Verweij PE, Brüggemann RJM, Azoulay E (2021). Taskforce report on the diagnosis and clinical management of COVID-19 associated pulmonary aspergillosis. Intensive Care Med.

[5] Koehler P, Bassetti M, Chakrabarti A, European Confederation of Medical Mycology, International Society for Human Animal Mycology, Asia Fungal Working Group, INFOCUS LATAM/ISHAM Working Group, ISHAM Pan Africa Mycology Working Group, European Society for Clinical Microbiology, Infectious Diseases Fungal Infection Study Group, ESCMID Study Group for Infections in Critically Ill Patients, Interregional Association of Clinical Microbiology and Antimicrobial Chemotherapy, Medical Mycology Society of Nigeria, Medical Mycology Society of China Medicine Education Association, Infectious Diseases Working Party of the German Society for Haematology and Medical Oncology, Association of Medical Microbiology, Infectious Disease Canada (2021). Lancet Infect Dis.

[6] Mitaka H, Kuno T, Takagi H (2021). Incidence and mortality of COVID-19-associated pulmonary aspergillosis: A systematic review and meta-analysis. Mycoses.

[7] Prattes J, Wauters J, Giacobbe DR (2022). ECMM-CAPA Study Group. Risk factors and outcome of pulmonary aspergillosis in critically ill coronavirus disease 2019 patients-a multinational observational study by the European Confederation of Medical Mycology. Clin Microbiol Infect.

[8] Salmanton-García J, Sprute R, Stemler J (2021). FungiScope European Confederation of Medical Mycology/The International Society for Human and Animal Mycology Working Group. COVID-19-Associated Pulmonary Aspergillosis, March-August 2020.. Emerg Infect Dis.

[9] Iqbal A, Ramzan M, Akhtar A (2021). COVID-Associated Pulmonary Aspergillosis and Its Related Outcomes: A Single-Center Prospective Observational Study. Cureus.

[10] Lahmer T, Kriescher S, Herner A (2021). Invasive pulmonary aspergillosis in critically ill patients with severe COVID-19 pneumonia: Results from the prospective AspCOVID-19 study. PLoS ONE.

[11] Beltrame A, Stevens DA, Haiduven D (2023). Mortality in ICU Patients with COVID-19-Associated Pulmonary Aspergillosis. J Fungi (Basel)..

[12] Calderón-Parra J, Mills-Sanchez P, Moreno-Torres V, HUPH IFI Study Group (2022). COVID-19-associated pulmonary aspergillosis (CAPA): Risk factors and development of a predictive score for critically ill COVID-19 patients. Mycoses.

[13] Xu J, Yang X, Lv Z (2021). Risk Factors for Invasive Aspergillosis in Patients Admitted to the Intensive Care Unit With Coronavirus Disease 2019: A Multicenter Retrospective Study. Front Med (Lausanne).

[14] Bartoletti M, Pascale R, Cricca M, PREDICO Study Group (2021). Epidemiology of Invasive Pulmonary Aspergillosis Among Intubated Patients With COVID-19: A Prospective Study. Clin Infect Dis.

[15] Hurt W, Youngs J, Ball J et al. COVID-19-associated pulmonary aspergillosis in mechanically ventilated patients: a prospective, multicentre UK study. Thorax. 2023 Sep 1:thorax-2023–220002. 10.1136/thorax-2023-220002. 10.1136/thorax-2023-220002 PMC10804023 37657925

[16] Chong WH, Saha BK, Neu KP (2022). Comparing the clinical characteristics and outcomes of COVID-19-associate pulmonary aspergillosis (CAPA): a systematic review and meta-analysis. Infection.

[17] Hong W, White PL, Backx M (2022). CT findings of COVID-19-associated pulmonary aspergillosis: a systematic review and individual patient data analysis. Clin Imaging.

[18] Fischer T, El Baz Y, Graf N (2022). Clinical and Imaging Features of COVID-19-Associated Pulmonary Aspergillosis. Diagnostics (Basel).

[19] Hashim Z, Neyaz Z, Marak RSK (2022). Practice Guidelines for the Diagnosis of COVID-19-Associated Pulmonary Aspergillosis in an Intensive Care Setting. J Intensive Care Med.

[20] White PL, Dhillon R, Cordey A (2021). A National Strategy to Diagnose Coronavirus Disease 2019-Associated Invasive Fungal Disease in the Intensive Care Unit. Clin Infect Dis.

[21] Ranieri VM, Rubenfeld GD, ARDS Definition Task Force (2012). Acute respiratory distress syndrome: the Berlin Definition. JAMA.

[22] WHO. Clinical management of COVID-19. World Health Organization. Interim guidance 27 May, 2020. https://www.who.int/publications-detail/clinical-management-of-covid-19. Date last updated: 23 Nov 2021. Date last accessed: 2 Jan 2024.

[23] WHO. Clinical care of severe acute respiratory infections. https://www.who.int/publications/i/item/clinical-care-of-severe-acute-respiratory-infections-tool-kit. Date last updated: 6 Apr 2022. Date last accessed: 2 Jan 2024.

[24] Oliva A, CogliatiDezza F, Petrucci F (2023). Outcome of COVID-19 patients with haematological malignancies after the introduction of vaccination and monoclonal antibodies: results from the HM-COV 2 0 study. Clin Exp Med..

[25] Oliva A, Ceccarelli G, Borrazzo C (2021). Comparison of clinical features and outcomes in COVID-19 and influenza pneumonia patients requiring intensive care unit admission. Infection..

[26] Horby P, Lim WS, Recovery Collaborative Group (2021). Dexamethasone in Hospitalized Patients with Covid-19. N Engl J Med.

[27] Hashim Z, Nath A, Khan A (2022). New insights into development and mortality of COVID-19-associated pulmonary aspergillosis in a homogenous cohort of 1161 intensive care patients. Mycoses.

[28] Patterson TF, Thompson GR, Denning DW (2016). Practice Guidelines for the Diagnosis and Management of Aspergillosis: 2016 Update by the Infectious Diseases Society of America. Clin Infect Dis.

[29] Bulpa P, Dive A, Sibille Y (2007). Invasive pulmonary aspergillosis in patients with chronic obstructive pulmonary disease. Eur Respir J.

[30] Salazar F, Bignell E, Brown GD (2022). Pathogenesis of Respiratory Viral and Fungal Coinfections. Clin Microbiol Rev.

[31] Apostolopoulou A, Clancy CJ, Skeel A (2021). Invasive Pulmonary Aspergillosis Complicating Noninfluenza Respiratory Viral Infections in Solid Organ Transplant Recipients. Open Forum Infect Dis..

[32] Dimopoulos G, Almyroudi MP, Myrianthefs P (2021). COVID-19-Associated Pulmonary Aspergillosis (CAPA). J Intensive Med.

[33] Tavakoli M, Shokohi T, Lass Flörl C (2020). Immunological response to COVID-19 and its role as a predisposing factor in invasive aspergillosis. Curr Med Mycol.

[34] Chen G, Wu D, Guo W (2020). Clinical and immunological features of severe and moderate coronavirus disease 2019. J Clin Invest..

[35] Crum-Cianflone NF (2016). Invasive Aspergillosis Associated With Severe Influenza Infections. Open Forum Infect Dis..

