# Supplementary material for: Risk factors for COVID-19 associated pulmonary aspergillosis and outcomes in patients with acute respiratory failure in a respiratory sub-intensive care unit
Source: BMC Infect Dis. 2024 Apr 11;24:392. doi: 10.1186/s12879-024-09283-3 (PMC11007928; doi:10.1186/s12879-024-09283-3)
Supplement: Supplementary file 1 — Supplementary Material 1. [file 12879_2024_9283_MOESM1_ESM.docx]

**Supplementary Table1. Clinical and mycological features of CAPA cohort**

|  | Age>65 (years) | APACHE II * | CCI * | P/F* | FIO2 * (%) | Hematological malignancy | Transfer to ICU | Lenght of stay (days) | Aspergillus coltural positive | GM index  (TBA) | GM index (serum) | Repeated chest CT with specific patterns | CAPA  classification | Death |
| --- | --- | --- | --- | --- | --- | --- | --- | --- | --- | --- | --- | --- | --- | --- |
| Sub. 01 | yes | 15 | 7 | 155 | 60 | no | no | 60 | no | 1.5 | 1.6 | yes | Possible | no |
| Sub. 02 | yes | 13 | 5 | 165 | 60 | no | no | 44 | no | 3.8 ** | NP | yes | Possible | no |
| Sub. 03 | yes | 20 | 8 | 386 | 21 | no | no | 54 | no | 3.4 ** | NP | yes | Possible | no |
| Sub. 04 | no | 11 | 6 | 300 | 21 | no | no | 58 | yes | 10 | 10 | yes | Probable | no |
| Sub. 05 | yes | 11 | 8 | 170 | 40 | no | no | 56 | no | 7.7 | NP | yes | Possible | no |
| Sub. 06 | no | 5 | 2 | 273 | 45 | no | no | 18 | no | NP | 1.7 | no | Probable | no |
| Sub. 07 | yes | 5 | 4 | 103 | 60 | no | no | 69 | no | 1.9 ** | NP | no | Possible | no |
| Sub. 08 | yes | 11 | 5 | 258 | 50 | no | no | 30 | no | 6.5 | NP | no | Probable | no |
| Sub. 09 | no | 11 | 3 | 323 | 21 | no | no | 93 | no | 10 | NP | yes | Possible | no |
| Sub. 10 | yes | 18 | 10 | 164 | 50 | no | no | 108 | no | 7.1 | NP | no | Possible | no |
| Sub.11 | yes | 6 | 5 | 116 | 80 | no | no | 38 | yes | 4.9 ** | NP | no | Possible | no |
| Sub. 12 | yes | 17 | 11 | 198 | 21 | no | no | 54 | no | 8.1 | NP | yes | Possible | no |
| Sub. 13 | no | 13 | 6 | 329 | 21 | no | no | 67 | yes | 8.1 | NP | no | Probable | no |
| Sub. 14 | yes | 24 | 8 | 433 | 21 | no | no | 24 | no | 2.5 ** | NP | no | Possible | no |
| Sub. 15 | yes | 13 | 3 | 210 | 60 | yes | no | 47 | no | 4 ** | NP | yes | Possible | no |
| Sub. 16 | no | 12 | 5 | 109 | 90 | yes | no | 82 | no | 1.7 ** | NP | yes | Possible | no |
| Sub. 17 | no | 7 | 4 | 270 | 40 | yes | no | 27 | no | NP | 1.8 | yes | Possible | no |
| Sub. 18 | no | 10 | 6 | 362 | 21 | yes | yes | 89 | no | 6.5 | NP | yes | Probable | no |
| Sub. 19 | yes | 6 | 6 | 367 | 21 | yes | no | 22 | no | 1.7 ** | NP | yes | Possible | no |
| Sub. 20 | yes | 9 | 8 | 238 | 40 | yes | no | 23 | no | 4.58 ** | NP | yes | Possible | no |
| Sub. 21 | yes | 6 | 7 | 385 | 28 | yes | no | 35 | yes | 0.9 *** | NP | yes | Probable | no |
| Sub. 22 | yes | 9 | 6 | 287 | 60 | no | yes | 21 | yes | NP | NP | no | Possible | yes |
| Sub. 23 | no | 11 | 3 | 143 | 60 | no | no | 39 | no | 3.7 ** | NP | no | Possible | yes |
| Sub. 24 | no | 15 | 7 | 339 | 28 | no | yes | 16 | no | 0.4 ** | 0.8 | no | Possible | yes |
| Sub. 25 | yes | 22 | 7 | 165 | 40 | no | no | 21 | no | 6.6 | NP | no | Possible | yes |
| Sub. 26 | yes | 12 | 9 | 197 | 35 | no | no | 11 | no | 3.4 ** | NP | no | Possible | yes |
| Sub. 27 | yes | 7 | 4 | 123 | 60 | no | no | 10 | no | 2.7 ** | 0.6 | no | Probable | yes |
| Sub. 28 | no | 7 | 5 | 482 | 28 | no | no | 64 | no | 2.5 ** | NP | no | Probable | yes |
| Sub. 29 | yes | 13 | 12 | 219 | 31 | no | no | 48 | no | 2.3 ** | NP | yes | Probable | yes |
| Sub. 30 | yes | 21 | 8 | 293 | 60 | no | no | 19 | yes | NP | NP | yes | Possible | yes |
| Sub. 31 | yes | 12 | 4 | 305 | 40 | no | no | 40 | yes | 5 | NP | no | Probable | yes |
| Sub. 32 | yes | 19 | 9 | 103 | 60 | no | no | 9 | no | 2.5 ** | NP | no | Possible | yes |
| Sub. 33 | yes | 15 | 11 | 207 | 60 | no | no | 65 | yes | 1.5 *** | NP | yes | Probable | yes |
| Sub. 34 | yes | 13 | 4 | 118 | 50 | no | yes | 19 | no | 1.8 ** | NP | no | Possible | yes |
| Sub. 35 | yes | 10 | 5 | 153 | 85 | no | no | 15 | no | 3.8 ** | NP | no | Possible | yes |
| Sub. 36 | yes | 10 | 6 | 110 | 60 | no | no | 10 | no | 10 | NP | no | Probable | yes |
| Sub. 37 | yes | 25 | 6 | 66 | 100 | no | no | 20 | yes | 2.3 | NP | no | Probable | yes |
| Sub. 38 | yes | 14 | 10 | 380 | 21 | no | no | 33 | no | 3.5 * | 0.5 | yes | Possible | yes |
| Sub. 39 | yes | 9 | 4 | 267 | 21 | no | no | 23 | yes | 6.5 | NP | yes | Possible | yes |
| Sub. 40 | yes | 20 | 5 | 252 | 21 | no | no | 9 | no | 7.3 | NP | no | Possible | yes |
| Sub. 41 | yes | 25 | 9 | 105 | 60 | no | no | 14 | yes | 6.5 | NP | yes | Possible | yes |
| Sub. 42 | yes | 19 | 10 | 201 | 70 | no | no | 63 | yes | 1.9 | NP | yes | Probable | yes |
| Sub. 43 | yes | 14 | 6 | 286 | 35 | yes | no | 3 | no | 1.8 ** | NP | no | Possible | yes |
| Sub. 44 | yes | 9 | 5 | 304 | 28 | yes | yes | 29 | no | 4.2 ** | 0.5 | yes | Possible | yes |
| Sub. 45 | yes | 17 | 5 | 106 | 100 | yes | no | 27 | no | 3.8 ** | 0.9 | yes | Possible | yes |
| Sub. 46 | yes | 25 | 8 | 332 | 31 | yes | no | 16 | no | NP | 3.4 | no | Probable | yes |
| Sub. 47 | yes | 12 | 8 | 263 | 35 | yes | no | 47 | yes | 6.9 | NP | yes | Possible | yes |
| Sub. 48 | yes | 10 | 5 | 265 | 40 | yes | no | 48 | yes | 6.1 | NP | yes | Possible | yes |
| Sub. 49 | yes | 7 | 4 | 338 | 21 | yes | yes | 71 | no | 2.6 ** | NP | yes | Possible | yes |
| Sub. 50 | yes | 29 | 8 | 108 | 100 | yes | no | 28 | no | 1.3 ** | NP | no | Possible | yes |
| Sub. 51 | yes | 11 | 11 | 329 | 28 | yes | no | 30 | no | 10 | NP | no | Possible | yes |
| Sub. 52 | yes | 17 | 2 | 295 | 21 | yes | no | 11 | no | 5.6 | 2 | yes | Probable | yes |
| Sub. 53 | no | 12 | 3 | 343 | 21 | yes | no | 63 | no | 6.5 | NP | yes | Possible | yes |
| Sub. 54 | yes | 15 | 5 | 148 | 50 | yes | no | 10 | yes | NP | NP | no | Possible | yes |
| Sub. 55 | yes | 14 | 5 | 291 | 35 | yes | no | 41 | no | NP | 5.7 | no | Probable | yes |
